# Bidirectional Crosstalk between the Heart and Brain in Alzheimer's Disease

**DOI:** 10.14336/AD.2024.1132

**Published:** 2025-10-31

**Authors:** Zhitian Wang, Lv Zhou, Na Zhao, Zhijun Zhang, Junjian Zhang, Qing-Guo Ren

**Affiliations:** ^1^Department of neurology, Zhongda Hospital, School of Medicine, Jiangsu Provincial Key Laboratory of Brain Science and Medicine, Southeast University, Nanjing 210009, China.; ^2^Zhongnan Hospital of Wuhan University, Wuhan, Hubei 430071, China.

**Keywords:** Alzheimer’s disease;, cardiovascular system, heart, brain, crosstalk, intervention strategies

## Abstract

Alzheimer's disease (AD) is a neurodegenerative disorder condition linked to various systemic comorbidities. Numerous studies have shown bidirectional crosstalk between the heart and the brain, but the specifics of how these interactions occur in AD are poorly understood. This narrative review summarizes the clinical evidence for a firm link between AD and cardiovascular health and discusses the bidirectional roles of AD and the cardiovascular system. AD pathogenic proteins, AD risk genes, neurohormones, the autonomic nervous system, and neurotransmitters may affect cardiovascular health, and cardiac-derived proteins, neurohormones, vascular function, inflammation, and other potential specific molecules or neural pathways may also influence AD pathology and cognitive function. Additionally, we propose potential AD intervention strategies based on the heart-brain axis to provide novel insights into AD prevention and treatment.

## 1. Introduction

Alzheimer's disease (AD) is the most prevalent form of dementia among the elderly, clinically characterized by a progressive decline in memory and cognitive function [1, 2]. AD patients suffer from loss of independent living ability in the late stages of life, and the cost of care is very high, which creates a huge health and economic burden on individuals, families, and society [3]. Plaques formed by amyloid-beta (Aβ) deposits and neurofibrillary tangles (NFTs) created by aggregates of hyperphosphorylated tau protein are the primary pathological features of AD [4]. AD has traditionally been discussed as a neurodegenerative disease of the brain. However, numerous studies in recent years have demonstrated that abnormalities in peripheral organs and tissues also lead to brain AD-type pathological changes and cognitive impairment [5-11]. Clinical studies have also shown that AD and dementia risk are associated with systemic comorbidities [12-15]. Systemic factors from the peripheral circulation can enter the brain and mediate neurodegeneration and AD pathogenesis [16-18]. Thus, AD is not just a single brain disease, but also has complex interactions with organs and tissues throughout the body. Exploring the mechanisms by which the peripheral system affects the onset and progression of AD can provide new perspectives for developing new prevention and treatment strategies [19].

The cardiovascular system is a completely closed blood circulation tube consisting of the heart and blood vessels. It takes the heart as its center of motion and transports blood through the blood vessels to all organs and tissues of the body to supply oxygen, nutrients, and other important substances to maintain the normal physiological activities of the organism [20]. The heart is directly regulated primarily by basic nerve centers located within the pons and medulla oblongata. A variety of central neurotransmitters (e.g., glutamate, acetylcholine, histamine) and neurohormones (e.g., adrenocorticotropic hormone, angiotensin, estrogen), as well as their intracellular signaling pathways, are also involved in regulating cardiovascular activity [21-24]. Weakened heart pumping leads to decreased blood flow to the brain and neurohormonal activation, subsequently causing a lack of nutrient supply and damage to neurons [25]. Clinical findings indicate that individuals with AD have a higher risk of cardiovascular disease, causing impairment of cardiovascular function, particularly diastolic function [26-28]. The connection between AD and cardiovascular conditions in both mice and humans has now been reported, emphasizing the interaction between the heart and brain in AD [29-32]. In this review, we summarize the clinical evidence linking cardiovascular disease and AD and explore the impact of AD on cardiovascular function and its underlying mechanisms. Moreover, we also discuss potential mechanisms by which the heart affects the pathogenesis of AD, including those involving cardiac-derived proteins, vascular dysfunction, inflammation, and other potential specific molecules or neural pathways. Based on the crosstalk between the heart and brain, we propose some heart-based intervention strategies for AD prevention and treatment.

**Table 1:** Clinical studies into the links among cardiovascular disease, cognitive impairment, and AD pathology.

Study	Type	Participants	Variables	Correlations
**[36]**	Cohort study	103869 Danes	Risk of developing dementia	Positive
**[37]**	Cross-sectional study	1625 Germans	Mild cognitive impairment	Positive
**[38]**	Mendelian randomization study	63926 Europeans	Risk of developing AD	Negative
**[40]**	Prospective Cohort study	457204 participants from UK Biobank	Dementia diagnosis	Positive
**[41]**	Observational study	53 Turks	Altered diastolic function, aortic stiffness	Positive
**[42]**	Observational study	25 Americans	Tau proteins	Positive
**[43]**	Cross-sectional study	306 Americans	Cerebral Aβ	Positive
**[44]**	Cohort study	635 Dutch	Aβ pathologies	Positive

Abbreviations: AD, Alzheimer’s disease; Aβ, amyloid beta.

## 2. Clinical evidence of associations between cardiovascular disease and AD

Recently, a mendelian randomization analysis of large-scale cardiovascular disease and AD genetic datasets has shown a causal relationship between cardiovascular disease and AD [33]. Coronary artery disease (CAD) is a widespread disorder of the cardiovascular system in which atherosclerosis of the coronary arteries causes narrowing or occlusion of the lumen of the arteries, leading to hypoxia-ischemia or necrosis of the myocardium [34, 35]. A cohort study showed that CAD patients had a significantly increased risk of AD, vascular dementia, and all-cause dementia than controls [36]. Cross-sectional studies indicate that elevated levels of NT-proBNP, a marker of cardiac stress, are linked to mild cognitive impairment [37]. Subsequent studies and meta-analyses further confirmed a significantly increased risk of cognitive decline in patients with heart failure (HF) [38-40]. Conversely, AD is also a risk factor for cardiovascular disease. A large of clinical evidence suggests that AD patients are at increased risk for cardiovascular disease, causing impairment of cardiovascular function, particularly diastolic function [41, 42]. AD patients also show a significant age-dependent increase in left ventricular wall thickness [43]. A study from Turkey investigating 29 AD patients and 24 age-matched normal controls found that AD patients were more likely to have diastolic dysfunction, higher arterial stiffness, and impaired blood flow transfer efficiency. These findings suggest that changes in the brain of AD patients might affect heart functions. A longitudinal study showed that hypertensive patients had a 4.8-fold higher risk of developing dementia, along with a greater number of Aβ plaques in the cortex and hippocampus, as well as a significant rise in tau deposition [44].In summary, a large amount of clinical evidence has associated cardiovascular disease with AD (Table 1).

## 3. Effects of AD on cardiovascular function

The brain is the command center of systemic tissues and organs[45]. The brain has been shown to modulate cardiovascular activity through neuromodulation and humoral regulation, and to affect cardiovascular function through chemical and electronic signals[46]. AD patients mainly exhibit brain atrophy, neurotransmitter imbalance, and neural circuit damage[47-49]. In clinical cohort studies, AD patients had significant decreases in cardiac ejection fraction and cerebral blood flow several years after diagnosis, along with an increase in plaque buildup in the aorta and carotid arteries [50]. Thus, neuropathological changes in the AD brain may lead to cardiovascular dysfunction.

### 3.1 Effect of AD pathogenic proteins on heart

Aβ peptides are generated through the hydrolysis of amyloid precursor protein (APP). Studies have found APP and Aβ are present in various tissues and organs other than the central nervous system (CNS), such as bone, muscle, gut, and heart [51]. Aβ may be associated with pathologies beyond AD, such as atherosclerosis. Aβ was found to promote endothelial cell activation and upregulate the expression of low-density lipoprotein receptor (LDLR) and low-density lipoprotein receptor-related protein 1 (LRP1) on the cell membrane, resulting in significant lipid accumulation in the intima of coronary arteries. After lipid aggregation damaged endothelial cells, Aβ triggered platelet aggregation and degranulation, which further advanced the progression of atherosclerotic plaques or caused micro-thrombotic events, ultimately leading to coronary atherosclerosis [52-54]. Intracellular accumulation of Aβ40 in endothelial and cardiomyocytes had also recently been found to be a direct cause of cytotoxicity, with changes in the transcriptional profiles of Aβ40-exposed endothelial and cardiomyocytes, in particular the up-regulation of genes associated with the ubiquitin-proteasome system, apoptosis, DNA damage, and inflammation [55, 56]. In addition, Aβ40 is thought to be associated with vascular aging, and accumulation of Aβ40 in the blood and heart tissue has been linked to poor outcomes in patients with cardiac insufficiency, CAD, and HF [57-60]. A subsequent study from Rotterdam of 4,156 controls and HF patients with plasma Aβ40 samples collected between 2002 and 2005, showed that higher Aβ40 levels were associated with poorer left ventricular ejection fraction (LVEF), a larger left ventricle, and a higher risk of HF [61]. A subsequent basic study demonstrated that 12-month-old Tg2576 mice had reduced LVEF and fraction shortening percentage compared with age-matched WT littermates. The hearts of Tg2576 mice demonstrated a buildup of amyloid aggregates, including Aβ, along with heightened interstitial fibrosis and pronounced cardiac neurological impairment [62]. In addition to Aβ plaques, NFTs consisting of hyperphosphorylated tau proteins are also key pathological characteristics of AD. Studies have indicated that genetic variation in tau may contribute to age-related systemic amyloidosis and increase the risk of myocardial infarction (MI), suggesting a link between tau and cardiac amyloidosis. The researchers also detected tau protein in ultrastructural examinations of heart tissue biopsies taken from MI patients [63]. Compared to younger mice, tau-deficient mice aged 23 months exhibited cardiac hypertrophy, a significant reduction in left atrial contractility, elevated blood pressure, and heightened sensitivity of isolated mesenteric arteries to angiotensin II (Ang II) contraction and isoprenaline relaxation [64]. The above suggests that both common pathological hallmarks of AD are involved in regulating cardiovascular function.

### 3.2 Effect of AD causative and risk genes on heart

Autosomal dominant disorders caused by APP, presenilin 1 (PSEN1), and presenilin 2 (PSEN2) are regarded to result in early-onset familial AD. Mutations in these genes influence the processing and removal of Aβ [65, 66]. The Swedish mutation of APP (APPswe) could lead to early-onset AD [67]. APPswe could cause cardiomyocyte contractile dysfunction and HF, indicating that AD and HF may have similar pathogenic mechanisms [68]. Notably, PSEN1 and PSEN2 not only function in the brain, but are also present in the heart, where they are essential for cardiac development. A case-control study found that subjects with PSEN1 mutations leading to early onset of AD had a markedly elevated risk of cardiovascular disease [69]. Early studies have indicated that polymorphisms in the PSEN1 gene are linked to susceptibility to dilated cardiomyopathy (DCM). Although a cross-sectional survey in China found no association between the PSEN1 gene and genetic variants in patients with sporadic DCM [70], other studies noted that Aβ aggregation and PSEN1 gene variants were found in the heart tissue of patients with idiopathic DCM [71]. Furthermore, PSEN1 expression was found to be downregulated in ischemia-induced HF, and PSEN1-deficient mice exhibited significant reductions in ejection fraction and fractional shortening percentage, suggesting that loss of PSEN1 may cause cardiac disease by interfering with calcium homeostasis [72]. RNA-seq analysis revealed an association between hypertrophic cardiomyopathy and PSEN2 gene variants [73]. In summary, AD-causing genes including APP and PSEN1/2 are involved in the development of cardiovascular diseases, and their mechanisms need to be further investigated.

In addition, AD has multiple genetic risk factors, and apolipoprotein E (APOE) is the risk gene that has been confirmed to be most strongly associated with the risk of late-onset AD [74, 75]. APOE ε4 raised the risk of AD in a dose-dependent manner by enhancing toxicity and inhibiting protective function [76]. In a prospective general population cohort study involving approximately 91,000 subjects, APOE levels were linked to a higher risk of ischemic heart disease [77]. A meta-analysis of several studies in Asian populations showed that carriers of the APOE ε4 gene had a 42% increased risk of CAD [78]. In another study, APOE ε4 was linked to cardiac remodeling and a higher risk of HF. APOE ε2 conferred stronger contractility to cardiomyocytes and enhanced heart function [79]. In addition, the occurrence of the APOE ε4 allele was notably higher in DCM patients, suggesting that this allele may also serve as a novel risk factor for non-ischemic cardiomyopathy [80]. In animal models, APOE-deficient mice have increased microvascular and endocardial damage, along with a faster progression of atherosclerosis compared with WT mice [81]. One of the key roles of APOE is to regulate cholesterol balance and facilitate the clearance of lipoproteins from the bloodstream. APOE gene deletion leads to elevated intracellular cholesterol and excessive accumulation of neutral lipids in cardiac lipid droplets, which may cause changes in levels of proteins such as adipose triglyceride lipase and ferritin, ultimately resulting in myocardial dysfunction [82]. APOE-deficient mice developed recurrent inflammatory and apoptotic processes that reduce antioxidant activity, increase cell proliferation or excessive intracellular lipid accumulation, and cause alterations in both the structure and function of the heart, thereby inducing cardiomyocyte damage and atherosclerosis [83]. It has also been shown that APOE signaling is associated with myocarditis pathology and that inhibition of APOE signaling reduces the pathological progression of myocarditis [84]. The complex role played by polymorphisms in the APOE gene highlights the plausible link between AD and cardiovascular from several perspectives.

Based on the above findings, AD-related pathogenic genes and risk genes, including APP, PSEN1/2, and APOE gene polymorphism, are all involved in the occurrence and development of cardiovascular diseases, revealing the intricate connection between AD and cardiovascular health.

### 3.3 Neurohormonal effects on the heart

It regulates blood pressure and fluid balance through the coordinated actions of the kidneys, cardiovascular, and central nervous systems. In HF patients, renal hypoperfusion activates the RAS due to reduced cardiac output and insufficient arterial filling, as well as direct adrenergic stimulation. Ang II levels are also elevated, facilitating a variety of pathophysiological processes [85-87]. Ang II increased the incidence of arrhythmias, especially during ischemia/reperfusion, and blockade of the Ang II type 1 receptor (AT1R) reduced the incidence of arrhythmias as well as mortality after MI [88]. Ang II is directly or indirectly associated with cardiac remodeling. Studies using cardiomyocytes in vitro and animal models in vivo demonstrated Ang II increased oxidative stress, leading to myocardial tissue remodeling, fibrosis, and left ventricular dysfunction [89, 90]. In addition, Ang II-induced hemodynamic changes promote systemic vasoconstriction and fluid retention, thereby exerting hemodynamic stress on the heart and leading to pathological cardiac remodeling [91]. Ang II is also a neurohormone with pro-inflammatory effects that contribute to vascular dysfunction through a variety of mechanisms, including its effects on endothelial cell function, as well as vascular inflammation and remodeling [92]. Ang II was able to trigger an inflammatory response by activating the nuclear factor κB (NF-κB) inflammatory pathway as well as resulting in an increase in the pro-inflammatory factors such as tumor necrosis factor α (TNF-α), interleukin 1 (IL-1), and IL-6 [93, 94]. Ang II downregulated endothelial nitric oxide synthase (eNOS) expression and lowered nitric oxide (NO) production, inducing endothelial dysfunction and vascular remodeling [95].

Several studies have shown a higher prevalence of CAD in perimenopausal and menopausal women [96-98]. Estrogen reduces the risk of atherosclerosis by improving endothelial function and inhibiting the recruitment of inflammatory cells. Reduced estrogen levels can lead to increased levels of certain inflammatory cytokines (e.g., TNF-α, IL-1, and IL-6) and altered immune cell profiles (e.g., increased T-cells), which ultimately affect cardiovascular function [99]. Studies have also demonstrated that estrogen-deficient mice exhibited oxidative stress, disturbed lipid metabolism, and other changes that accelerate atherosclerosis [100]. Estrogen is also closely associated with angiogenesis, promoting endothelial cell proliferation, migration, and differentiation, and maintaining the functional integrity of endothelial cells [101, 102]. In addition, other hormones, such as cortisol, also participate in the physiological regulation of the heart. Neurohormones regulate heart function by affecting myocardial contractility, heart rate, vascular tone, and fluid balance. Understanding the mechanisms of these hormones in the heart is crucial for developing treatment strategies for cardiovascular diseases.

### 3.4 Regulatory roles of peripheral nerves and neurotransmitters on the heart in AD

CNS regulates cardiac activity primarily through two divisions of the autonomic nervous system: the sympathetic and parasympathetic nervous systems [103]. Clinical evidence suggests that in the early stages of AD, patients experience autonomic dysfunction even before the onset of overt dementia symptoms, most commonly postural hypotension, decreased heart rate variability, constipation, urinary incontinence, and syncope [104]. Early AD mice show marked abnormalities in autonomic function, mainly in the form of enhanced sympathetic hyperactivity and inhibition of parasympathetic activity. With aging, degenerative changes in autonomic nerves also appear in peripheral nerves. In the cardiovascular system, excessive activation of the sympathetic nervous system induces over-release of adrenaline and noradrenaline from sympathetic nerve fibers, ultimately causing elevated levels of catecholamines in circulation, activating widespread adrenergic receptor groups on cardiac muscle cells, specifically β-adrenergic receptors (βARs). Prolonged activation of βAR induced cardiomyocyte dysfunction, apoptosis, and arrhythmias [105]. Transgenic mice with cardiac-specific overexpression of βAR exhibited cardiomyocyte hypertrophy, fibrosis, and progressive HF [106]. Another study revealed that sympathetic nerves caused mitochondrial dysfunction and Ca^2+^ overload in mouse cardiomyocytes via βAR activation [107]. These findings indicate that sympathetic nervous system activation in AD patients may lead to myocardial damage by stimulating βAR.

The occurrence of AD is associated with the degeneration of cholinergic neurons in the brain, depletion of cholinergic neurotransmitters, and reduced activity of cholinesterase [108, 109]. In peripheral nerves, acetylcholine serves as the primary neurotransmitter of parasympathetic nerves. Cholinergic receptors include muscarinic acetylcholine receptors (mAChRs) and nicotinic acetylcholine receptors (nAChRs) [110]. Cardiomyocytes primarily express m2AChR [111, 112]. Acetylcholine interacts with m2AChR to prevent a range of cardiovascular conditions, including myocardial infarction and arrhythmias [112]. According to previous studies, cholinergic stimulation enhanced cardiac function through increased parasympathetic activity and suppressing ventricular remodeling caused by stress or Ang II in rats [112, 113]. Studies have found that pressure overload triggered cardiac hypertrophy, decreased parasympathetic function in cardiac tissue and reduced expression of m2AChR. Activation of m2AChR inhibited protein expression in the NLRP3/caspase-1/IL-1β signaling pathway in cardiomyocytes, thereby reducing endoplasmic reticulum stress and inflammation, maintaining mitochondrial homeostasis and improving cardiac hypertrophy [111, 114, 115]. m2AChR has a beneficial impact on improving cardiac function. Acetylcholine is involved in pathological alterations in both the brain and the heart.

Neurotransmitters are also regarded to be important in the brain-heart axis. Glutamate is the primary excitatory neurotransmitter in the CNS, playing crucial roles in memory, synaptic plasticity, and neuron development. However, excessive glutamate stimulation is also linked to neuronal cell death. Overactive glutamatergic signaling appears during the initial phases of AD pathology, thought to be mediated by reduced glutamate uptake. Reduced glutamate clearance and resulting excitotoxicity are widely recognized as pathological mechanisms in AD [116]. Glutamate release could predict sustained myocardial ischemia in rats, particularly enhanced release from ischemic regions of the heart [117, 118]. Preliminary experimental studies also indicated that glutamate could induce cardiomyocyte apoptosis and arrhythmias by activating N-methyl-D-aspartate (NMDA) receptors and inducing local Ca^2+^ release [119, 120]. Elevated glutamate levels may be associated with cardiac toxicity induced by oxidative stress. Additionally, other neurotransmitters such as serotonin and histamine are also involved in the physiological regulation of the brain and the heart [121, 122]. The effects of more neurotransmitters on the brain-heart axis warrant further exploration in the future.

## 4. Effects of cardiovascular function on AD

We have already discussed the potential effects of AD on the heart. Due to the higher incidence of AD in patients with cardiovascular diseases such as HF and CAD, we further investigated the potential impacts of cardiac hormones, vascular dysfunction, neurohormones, inflammation, and other potential specific molecules or neural pathways on the pathogenesis of AD and cognitive function.

## 4.1 Heart-derived hormones

### 4.1.1 Cardiac troponin

Cardiac troponin (cTn) consists of three subunits: troponin T (cTnT), troponin I (cTnI), and troponin C (cTnC). When damage to cardiomyocytes occurs, intracellular cTn is released into the bloodstream. cTn elevation reflects damage to cardiomyocytes. When exploring the link between cardiovascular health and AD, cross-sectional studies have revealed that links among cardiac markers, cognitive performance, and brain structural changes with aging [123]. In particular, multiple clinical studies have shown that elevated plasma cTn levels are linked to a faster rate of cognitive decline and a greater risk of developing dementia [124-126]. This is particularly evident in patients with structural damage to the brain's microvasculature, with elevated cTn levels detected in patients exhibiting the most severe white matter lesions [123, 127]. This implies that subclinical damage to the cerebrovascular system may lead to the release of cTn, which in turn impacts cognitive function and may be related to the progression of AD. The FINRISK study found that cTnI could serve as a predictor for the development of AD [128]. Transcriptomic analysis of mice showed that the highest- ranked up-regulated gene, cTnC, encoded a calcium-binding protein that modulates actin binding in neurons [129]. A recent study found that prolonged increases in cTn release may result in the release of intracellular calcium, and that leaking calcium ions after prolonged stimulation may cross the blood-brain barrier (BBB) and cause calcium overload, resulting in hippocampal atrophy and disruption of neuronal signaling associated with AD [130]. There are relatively few basic studies on the regulatory role of cTn in AD, which need to be further explored in the future [131-133].

### 4.1.2 Brain natriuretic peptide

Brain natriuretic peptide (BNP) belongs to a group of diuretic natriuretic peptides released by the heart, and consists of a polypeptide of 32 amino acid residues [134]. BNP initially exists in the form of pro-BNP, which is cleaved by enzymes into inactive NT-proBNP and active BNP. BNP promotes natriuresis, diuresis, and vasodilation and has beneficial effects in inhibiting the RAS axis, the sympathetic nervous system, and cardiac hypertrophy and fibrosis [135]. It is worth noting that BNP may have a crucial regulatory function in the development of AD. Elevated levels of BNP in the blood have been linked to cognitive impairment and changes in brain microstructure in multiple clinical studies [37, 136, 137]. In addition, a 14-year prospective study evaluated 7,158 subjects without memory impairment and found that increased BNP levels were independently associated with dementia risk, further emphasizing the importance of BNP in cognitive decline [138]. A Finnish cohort study with an 18-year follow-up period suggests that BNP may be a predictor of AD disease development [128]. Furthermore, animal experiments also provide insights into the potential effects of BNP on AD. For example, intracerebroventricular injection of BNP can lead to decreased expression of glial cell-derived neurotrophic factor (GDNF) by inhibiting the extracellular signal-regulated kinase (ERK) - cAMP response element-binding protein (CREB) signaling pathway, which is linked to learning and memory dysfunction in rats [139]. However, there is also evidence that BNP can improve neurological function in brain-injured mice by increasing cerebral blood flow and reducing neuroinflammatory responses [140]. Several of the above clinical and prospective studies have shown high levels of BNP are a high-risk factor for the development of cognitive impairment and dementia, which increases the credibility of the findings. However, observational studies often struggle to establish a clear causal relationship. Animal experiments support the neuroprotective effects of BNP, but the specific mechanisms in clinical studies still need further validation. The dual role of BNP in the brain reflects its complex mechanisms. This may be related to the regulatory pathways of BNP in different pathological environments, receptor activation patterns, and interactions with other neurotransmitters or signaling molecules. Currently, there is limited research on the effects of BNP on AD. Future studies should focus more on the specific mechanisms of BNP in AD and explore how to maintain its neuroprotective effects while mitigating its potentially harmful impacts.

### 4.1.3 Lactate dehydrogenase

Lactate dehydrogenase (LDH) is a tetramer with a molecular weight of 140-kDa, which consists of two subunits, A and B, to form five isozymes, among which LDH1 and LDH2 are widely expressed in cardiomyocytes, which is one of the most important indicators for observing cardiomyopathies [141]. LDH is an enzyme of the glycolysis pathway, which can catalyze inter-conversion between pyruvate and lactate, and is closely related to myocardial energy metabolism [142]. In addition, LDH is crucial for brain energy metabolism and memory formation [143]. Increased lactate levels and LDH expression in aging and AD patients indicate that LDH might be a significant contributor to cognitive decline. Overexpression of LDH in neurons and glial cells resulted in a significantly shorter lifespan, and downregulation of LDH resulted in a longer lifespan. Furthermore, LDH overexpression disrupted circadian motor activity rhythms and significantly increased neurodegenerative brain lesions[144]. Increased lactate and LDH promote the release of pro-inflammatory factors from microglia, and senescent microglia H3K18 lactylation enhances brain senescence and AD pathological phenotypes through the NF-κB signaling pathway. Treatment of senescent microglia with LDH inhibitors leads to a significant reduction in lactate levels, decreases the release of pro-inflammatory factors, and attenuates neurodegeneration and cognitive deficits in AD [145]. However, LDH does not play a single role in AD. Some studies have shown that LDH and lactate can also play a protective role. LDH overexpression in neurons reduced mitochondrial respiration and reactive oxygen species (ROS) production, ultimately reducing Aβ toxicity [146]. The increase of LDH in aging and AD patients indicates its potential clinical significance, but a causal relationship has not yet been fully established. The role of LDH in cells is complex, exhibiting both protective and damaging effects, which may lead to inconsistencies in research findings. Most studies have focused on short-term effects, with limited research on the role of LDH in the progression of long-term neurodegenerative diseases and its potential cumulative effects. Future studies should focus on further exploring the role of LDH in different and long-term pathological states and investigating its potential as a therapeutic target for AD.

### 4.1.4 Creatine kinase

Creatine kinase (CK) consists of two subunits, M and B, forming the CK-BB, CK-MB, and CK-MM isoenzymes. CK is a kinase that is vital for ATP regeneration, intracellular energy conversion, and muscle contraction [147]. Clinically, CK is one of the cardiac enzyme tests that are mainly used in the diagnosis of myocardial-related diseases. When CK levels are high, it is often indicative of myocardial damage [148]. CK facilitates the conversion of adenosine diphosphate (ADP) and phosphocreatine to adenosine triphosphate (ATP) and creatine, playing a crucial role in managing the energy supply for muscle contraction, especially in the heart, through the maintenance of relatively stable ATP concentrations. Pathological hypertrophy and myocardial remodeling are significantly connected to reduced ATP levels and CK-related responses [149]. At the same time, published research suggests that CK is transported from outside to inside the body across the BBB. Changes in CK levels can alter CNS function and impair ATP synthesis, which may affect the brain's overall health [150].CK contributes to cognitive function, the generation of dendrites, and cellular motility by generating substantial quantities of ATP during brain activation. A cross-sectional study found that CK levels were considerably lower in individuals with AD than in mildly cognitively impaired individuals, indicating that reduced CK levels might be related to severe cognitive impairment [151]. Another study showed that the brains of AD patients exhibited reduced CK activity and mitochondrial dysfunction [152]. Further studies have indicated that CK activity is diminished in the brains of AD patients, accompanied by mitochondrial dysfunction [153]. These findings imply that CK could be crucial in the progression of cognitive decline and dementia, acting as a protective factor, and elevating CK levels may be a protective measure against AD.

### 4.1.5 Growth differentiation factor 15

Growth differentiation factor 15 (GDF15) belongs to the transforming growth factor-β (TGF-β) protein superfamily. Serum GDF15 levels increase with age as well as with cellular stress and mitochondrial dysfunction [154]. GDF15 is currently identified as a new cardiac-derived endocrine hormone that regulates body growth and has local cardioprotective effects, possibly due to its autocrine/paracrine properties: antioxidant, anti-inflammatory, and anti-apoptotic[153]. GDF15 was found to be highly induced in cardiomyocytes after ischemia-reperfusion [155]. GDF15 was also induced during cardiac hypertrophy in a pressure overload mouse model, and its cardiac-specific overexpression protected the heart from the hypertrophic response [156]. GDF15 has a protective role in enhancing cardiovascular health. Moreover, GDF15 is involved in the regulation of AD progression. Plasma proteomic profiling showed that individuals with elevated GDF15 levels have a 2.32 times greater risk of developing dementia [157]. Several studies have shown that elevated circulating levels of GDF15 are linked to an increased risk of developing AD and other neurodegenerative disorders [158], and that exogenous recombinant GDF15 enhances the clearance of Aβ in cultured microglial cells [159]. Furthermore, studies performed on in vitro and in vivo mouse models of AD suggested that administration of recombinant GDF15 may have positive effects by enhancing the proliferation and migration of hippocampal stem cells, while the absence of GDF15 resulted in decreased proliferation and migration of these cells [160]. GDF15 may offer a potential therapeutic avenue for AD.

### 4.2 Effects of neurohormones on the brain

Cardiovascular diseases often lead to the activation of the RAS. Elevated levels of Ang II may be associated with pathological features of AD, such as Aβ [161]. In spontaneously hypertensive rats, RAS activation may also affect the brain's internal environment, subsequently influencing cognitive abilities [162]. The RAS was causally linked to hippocampal damage, and hyperactivation of the RAS in AD patients and mice caused tau phosphorylation and enhanced Aβ toxicity, ultimately leading to altered AD pathology and cognitive impairment [161, 163-165]. Ang II could act on AT1 on microglia to promote neuroinflammatory responses and reduce acetylcholine release, which was further involved in the onset and progression of AD [166]. Ang II overexpressing mice had enhanced ischemic brain damage, reduced cerebral blood flow, increased oxidative stress, and ultimately cognitive deficits [167]. In addition, a growing body of evidence established that drugs targeting the RAS, particularly AT1 blocker (ARB) and angiotensin-converting enzyme inhibitor (ACEI), could slow the onset and progression of AD [168-170]. Future research focusing on how RAS activation induced by cardiovascular diseases affects the brain's internal environment, inflammatory responses, and metabolism may provide new insights for the treatment of AD.

Cardiovascular diseases are often accompanied by a decrease in estrogen levels, particularly after menopause in women [171]. Estrogen supplementation therapy improves cardiovascular status in postmenopausal women [172]. Low estrogen levels are associated with an increased risk of AD, potentially accelerating the progression of AD by affecting neuronal survival and function [173]. Estrogen plays a neuroprotective role in the brain and is important for maintaining normal brain function [174]. Estrogen was involved in regulating beta-site amyloid precursor protein cleaving enzyme 1 (BACE1) activity, inducing APP degradation and reducing Aβ accumulation [175]. Additionally, estrogen enhanced synaptic plasticity by increasing synaptophysin levels within hippocampal neurons [176]. Changes in estrogen levels due to cardiovascular diseases may play an important role in AD. In-depth studies on the role of estrogen in neuroprotection, inflammation regulation, and AD pathology will provide new insights for future prevention and treatment strategies.

### 4.3 Vascular dysfunction

The brain and heart are linked via neurovascular and humoral pathways, and managing vascular risk factors can help lessen the impacts of cardiovascular disease and AD. Vascular dysfunction is an early sign of Aβ buildup, and together with structural changes in the cerebral microvascular system, it may play a role in the progression of AD [177]. Large artery stiffness, as a sign of vascular aging and cardiovascular disease, is a significant factor in cognitive decline. In a large study of participants without dementia, a correlation was found between increased aortic stiffness and mild cognitive impairment [178]. Studies have shown that chronic hypertension leads to endothelial hyperplasia of arteries, resulting in narrowing of the arterial lumen, increased resistance to blood flow, and insufficient perfusion to important functional areas of the brain. This perfusion deficiency is particularly evident in the prefrontal cortex and the anterior cingulate region, but is also reflected in the cortical motor area and hippocampus, seriously affecting the attention, executive function, psychomotor speed, and working memory of hypertensive patients [179]. Elderly hypertensive patients may suffer from hypotension due to improper use of antihypertensive drugs, resulting in insufficient cerebral blood perfusion, local tissue ischemia, and hypoxia, and decreased neuronal function, inducing changes in the white matter and grey matter of the brain, and ultimately affecting cognitive function. Damage to the white matter and cortical interconnections results in the blockage of connections between functional areas, affecting the transmission of information and leading to cognitive impairment [180]. Basic experiments have shown that chronic hypertension also accelerates the accumulation of Aβ in the brain and promotes the overproduction of ROS by endothelial cells, leading to neuronal necrosis [181]. Based on these findings, we speculate that vascular factors also play an important role in the crosstalk between the heart and brain in AD.

### 4.4 Inflammation

Inflammation is a typical feature of cardiovascular disease and AD. Cardiac inflammation initiates in cardiomyocytes as a reaction to both ischemic and non-ischemic stressors. During ischemic stress, the damage-associated molecular pattern (DAMP) released by cardiomyocytes activates Toll-like receptors (TLRs), leading to NF-κB activation and the release of proinflammatory factors [182]. In response to nonischemic stresses such as Ang II and pressure overload, calmodulin-dependent protein kinase-δ (CaMKIIδ) is activated in cardiomyocytes, triggering pro-inflammatory gene expression mediated by NF-κB. CaMKIIδ signaling also stimulates the activation of NLRP3 inflammasomes in cardiomyocytes by elevating mitochondrial ROS, leading to the production of active IL- 1β and IL-18 [183, 184]. The cytokines and chemokines generated in cardiomyocytes initiate cardiac inflammation by attracting macrophages and later T cells, resulting in fibrosis, detrimental cardiac remodeling, and HF. Similarly, in AD, inflammation-related signaling pathways, including the NF-κB pathway, the complement system, and pro-inflammatory factors are activated, and the concentrations of inflammatory factors in the tissues and body fluids of the body of AD patients are significantly elevated [4, 185]. Genome-wide association studies have identified several risk genes associated with AD are also correlated with inflammation [186, 187]. In general, inflammation is not only a common pathological process of cardiovascular disease and AD, but systemic inflammation may also be a common risk factor for both. Inflammatory mediators released by the heart not only affect the heart itself, but may also affect other organs, including the brain [188-190]. HF was able to induce increased bone marrow and splenic monocyte production, causing skeletal muscle inflammation and adipose tissue inflammation [191, 192]. Peripheral inflammation has been known to exacerbate AD pathology and lead to cognitive deficits, whereas rejuvenated peripheral immune cells with increased anti-inflammatory capacity help to reduce neuroinflammation in the brain and decrease neuronal damage [193]. Therefore, we speculate that cardiovascular disease may affect the development of AD by aggravating systemic inflammatory response. Inflammatory mediators produced during the development of HF may promote neuroinflammation and neurodegenerative pathology through activation of neuroglia, which may contribute to AD. However, there are limited relevant studies reported recently, and the common role of inflammation in cardiovascular disease and AD needs to be further explored.

### 4.5 Alternative mechanisms

Above, we summarized the potential impacts of cardiac hormones, vascular dysfunction, and inflammation on the pathogenesis of AD and cognitive function. The association between cardiovascular disease and AD is widely acknowledged, yet its molecular mechanisms remain unclear. A study published in 2023 in *Nature Neuroscience* indicated that activation of the adrenergic pathway during HF led to Ca^2+^ leakage regulated by ryanodine receptor (RyR) channels within neurons [194]. This leakage of endoplasmic reticulum Ca^2+^ further triggered abnormal neurotransmitter release, enhanced inflammatory responses, neuronal mitochondrial dysfunction, and oxidative stress reactions. Targeted use of RyR channel drugs could improve tau protein pathology and cognitive impairments in HF mice, maintaining normal brain metabolic activities. RyR channels may be a crucial molecular mechanism through which HF impacts AD pathology and cognitive impairment. Although the direct link between AD and the sympathetic nervous system has not been fully established, recent studies have revealed the regulatory influence of the sympathetic nervous system in cardiovascular disease and its potential impact on cognitive function. A study published in 2023 in *Science* highlighted significant reductions in pineal gland sympathetic axons in heart disease patients and mouse models, accompanied by inflammatory macrophage aggregation in superior cervical ganglia (SCG), fibrosis, and selective loss of pineal gland neurons, ultimately leading to sleep disorders [195]. Considering the close association between sleep disorders and cognitive decline, which may indirectly promote AD pathogenesis by disrupting neural plasticity and effective clearance of metabolic waste, this provides a new perspective on how cardiovascular disease contributes to AD development. Furthermore, a study published in Nature Communications reinforced this connection, confirming that superior cervical ganglionectomy (SCGx) improved conditions in sleep-deprived mice and alleviated myocardial ischemia-reperfusion injury [196]. These findings suggest that SCG not only plays a crucial relay role in communication between the heart and brain but also may have potential significance in preventing and treating brain diseases caused by heart disease. Future exploration should further focus on more molecular mechanisms, seeking potential intervention targets, and aiming to provide new strategies for preventing and treating AD from the perspective of the brain-heart axis. Overall, there is relatively limited research literature currently available on the regulatory role of the cardiovascular system in AD. According to several studies published in authoritative journals, the RyR channels and the SCG may be key mechanisms through which cardiovascular diseases influence AD pathology and cognitive impairment. In the future, further exploration of additional molecular mechanisms and identification of potential intervention targets should be pursued, aiming to provide new strategies for the prevention and treatment of AD from the perspective of the brain-heart axis.

## 5. Potential AD prevention and treatment strategies based on the heart-brain axis

### 5.1 Prevention and management of cardiovascular diseases

As mentioned earlier, AD often coexists with cardiovascular disease, which may promote brain AD pathology and heighten the risk of developing AD. Therefore, prevention and management of cardiovascular diseases such as hypertension and HF are critical. Lifestyle interventions are feasible and effective in preventing and reducing the incidence of cardiovascular disease and AD. The dietary approach to stop hypertension (DASH) diet is a diet based on the basic principles of eating more whole grain foods and vegetables, moderate intake of lean poultry and fish, and limiting salt intake. The DASH diet can enhance patients' physical and functional abilities by lowering blood pressure, body weight, and LDL cholesterol, while also improving heart function, arterial flexibility, exercise capacity, and overall quality of life [197-199]. DASH diet also has a positive impact on patients with HF [200]. Studies have found that high-sodium diets are linked to a higher risk of cerebrovascular disease and dementia. Epidemiological data reveals that long-term high-sodium diets are associated with stroke, dementia, and cerebral white matter damage. Studies on memory, health, and aging have shown a positive link between the DASH diet and cognitive function, with both the DASH diet and the Mediterranean diet positively associated with enhanced cognitive abilities [201]. A prospective study found that the Mediterranean diet and the DASH diet reduced the risk of AD [202]. A good diet has positive effects on both cardiovascular disease and AD. However, patient adherence to dietary plans is often low and requires the cooperation of both patients and their families. In clinical settings, healthcare professionals should create adaptive dietary plans tailored to individual patients, taking into account their nutritional needs and health status. By conducting comprehensive assessments and providing personalized dietary prescriptions, patients can be encouraged to improve their eating habits, thereby enhancing their overall health and reducing the risk of cardiovascular disease and AD. Additionally, regular monitoring and adjustment of dietary plans are crucial to ensure safety and effectiveness.

Additionally, there is evidence that sedentary behavior is negatively correlated with myocardial contractility [203, 204]. Moderate exercise improves cardiovascular function, enhances myocardial contractility, reduces myocardial oxygen consumption, improves coronary blood flow, and reduces the incidence of CAD [205, 206]. The increase in the duration of sedentary behavior in the elderly is also significantly correlated with the incidence of AD. Sedentary behavior affects protein homeostasis, mitochondrial function, intercellular communication, and other aspects that accelerate biological aging [207]. The link between cardiovascular disease and AD has underlined the importance of avoiding sedentary behavior and increasing physical activity in AD. Studies have shown that exercise training increases cardiac ejection fraction in AD mice and prevents an increase in mitral valve deceleration time, restores elastin and aortic wall integrity, and has beneficial effects on cardiac and aortic function and structure [208]. In addition, a meta-analysis also confirmed that aerobic exercise can increase hippocampus and grey matter volume, induce brain-derived neurotrophic factor production, and improve cognitive brain network connectivity, which improves overall cognitive function in AD patients [209]. Exercise offers significant benefits for cardiovascular health and the prevention of AD and is highly feasible. Healthcare professionals should develop adaptive exercise plans based on individual patient conditions, taking into account physical load and potential risks, conducting comprehensive assessments, and providing personalized exercise prescriptions. Additionally, combining education and support can effectively encourage patient participation in physical activity. Regular monitoring and adjustment of exercise plans are also essential to prevent exercise from placing excessive strain on the heart.

Furthermore, several anti-cardiovascular drugs have demonstrated significant potential in preventing and treating AD. ACEIs and ARBs control blood pressure and improve HF, but also seem to have great potential in AD treatment. A multicenter cohort study indicated that older adults using ACEIs experienced slower cognitive decline [210, 211]. ARBs and ACEIs lower the risk and progression of AD [165]. A meta-analysis showed that ACEI use was significantly linked to a lower risk of AD and cognitive impairment in older adults. Candesartan enhances cerebral blood flow, decreases infarct size, and improves stroke outcomes [212]. Similarly, losartan prevented BBB disruption and restored blood flow after inducing stroke [213]. Olmesartan was also linked to enhanced cognitive function and hippocampal synaptic plasticity [214]. Telmisartan reduced cognitive impairment, reduced Aβ deposition, and attenuated oxidative stress and neuroinflammation in hypertensive patients [215]. In vitro studies have also shown that ACEIs are involved in the degradation of Aβ peptides, leading to reduced Aβ deposition and accumulation [216]. Animal models revealed that Ang II increased Aβ peptide accumulation and deposition in AD mice, and ACEI use also ameliorated tau hyperphosphorylation and neuronal degeneration in older mice [217, 218]. Other drugs may also play a role in AD treatment and need to be further explored in the future. However, the high cost of medication, as well as potential adverse reactions and drug dependence, may increase both the economic and psychological burdens on patients. Healthcare professionals should develop adaptive medication treatment plans based on individual patient circumstances, considering their health status and potential risks. Additionally, regular monitoring and adjustment of the medication regimen are essential measures to ensure safety and effectiveness.

### 5.2 Other potential intervention strategies

This study proposes that the regulation of cardiac-derived protein levels may offer new directions for the treatment of AD. CK has potential neuroprotective effects, and increasing its levels may contribute to the prevention and treatment of AD. GDF15 has also shown potential in promoting cognitive function and warrants further investigation. Considering the antagonistic role of cardiac-derived proteins in the progression of AD, exploring methods to enhance protective cardiac-derived proteins and inhibit their detrimental effects may provide a new perspective for AD treatment. Additionally, vascular reconstruction techniques such as percutaneous coronary intervention (PCI) may positively impact AD by improving cerebral blood flow [219]. Systemic anti-inflammatory therapy may mitigate the promoting effects of inflammation on AD progression. Furthermore, the development of drugs targeting RyR channels and exploration of SCGx provide new avenues for applying the brain-heart axis in AD treatment. In summary, the regulation of cardiac-derived proteins, improvement of vascular factors, inflammation control, drug development targeting specific mechanisms, and SCGx all offer new strategies for intervening in AD, which urgently need to be explored for their potential applications in the future.

## 6. Conclusions and perspectives

**Figure 1.**
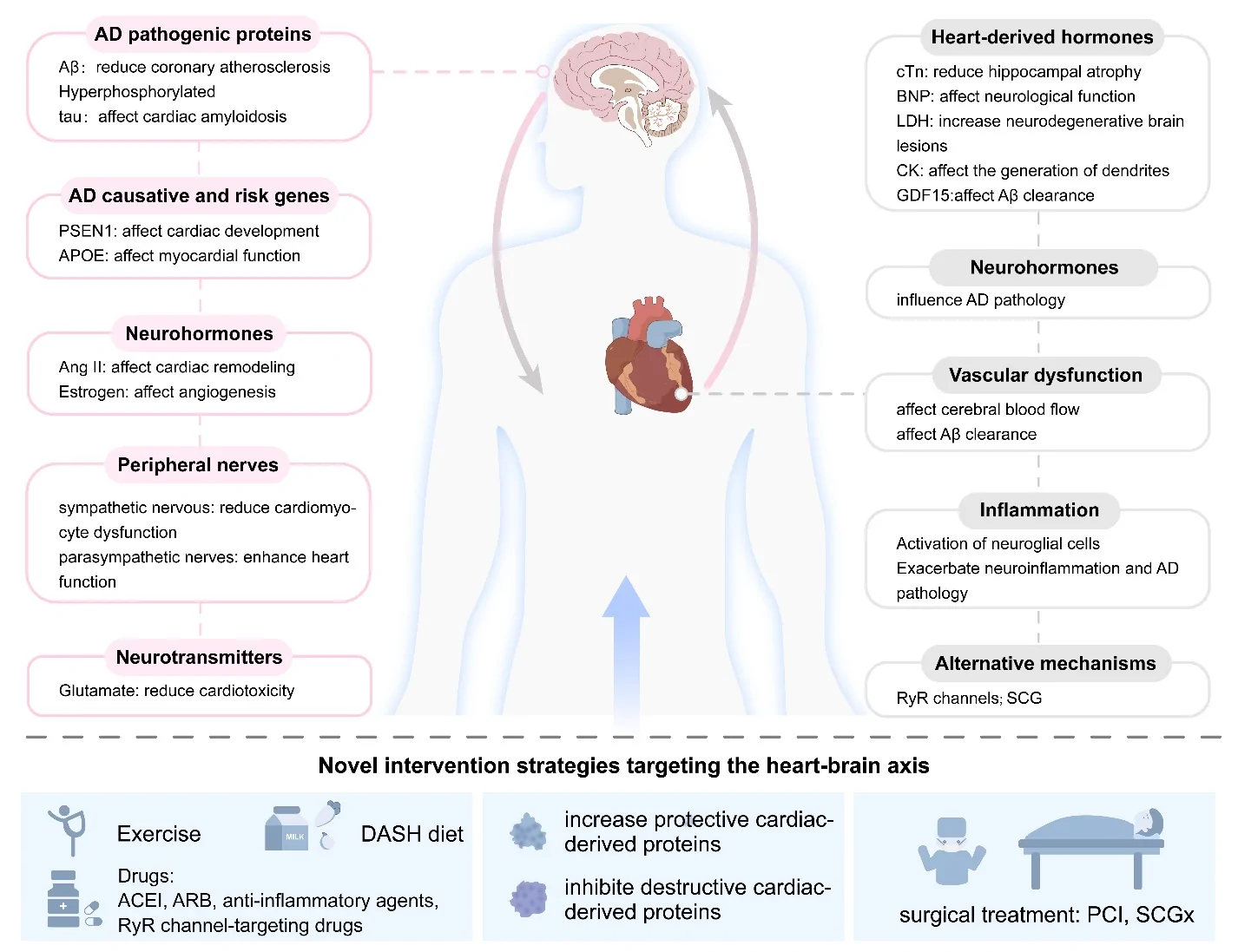
**Crosstalk between heart and brain in Alzheimer’s disease (AD)**. AD pathogenic proteins, AD risk genes, neurohormones, the autonomic nervous system and neurotransmitters may affect cardiovascular health, and cardiac-derived proteins, vascular function and inflammation may also influence AD pathology and cognitive function. Additionally, we propose potential AD intervention strategies based on the heart-brain axis with the aim of providing novel insights on AD prevention and treatment. Aβ, amyloid beta; APOE, apolipoprotein E; APP, amyloid precursor protein; AD, Alzheimer’s disease; Ang II, angiotensin II; BBB, blood-brain barrier; PSEN1, presenilin 1; cTn, cardiac troponin; BNP, brain natriuretic peptide; LDH, lactate dehydrogenase; CK, creatine kinase; GDF-15, growth differentiation factor 15; RyR, ryanodine receptor; SCG, superior cervical ganglia; SCGx, superior cervical ganglionectomy; PCI, percutaneous coronary intervention

This review not only provides an overview of the clinical evidence supporting a strong connection between AD and cardiovascular health, but also discusses the bidirectional roles of AD and cardiovascularity. AD-causing proteins, AD risk genes, neurohormones, the autonomic nervous system, and neurotransmitters may affect cardiovascular health, and cardiogenic proteins, neurohormones, vascular function, inflammation, RyR channels, and SCG may also influence AD pathology and cognitive function. In addition, age, gender, sedentariness, and dietary patterns may be common risk factors that link and promote the two diseases, cardiovascular disease and AD. The heart-brain axis plays a crucial role in the pathogenesis of AD, and its clinical significance cannot be overlooked. Understanding the interactions within the heart-brain axis can not only help identify high-risk patients but also provide healthcare professionals with opportunities for early intervention. In clinical practice, multidisciplinary teams should collaborate to develop comprehensive assessment plans that incorporate cardiovascular, endocrine, and immune considerations in patient management. Additionally, implementing lifestyle interventions and personalized treatment plans can further improve the overall health status of patients and slow the progression of AD. Overall, the mutual crosstalk between the heart and brain provides new perspectives on the treatment of AD and cardiovascular disease, which may interact and promote each other. Proactively preventing and treating cardiovascular disease in older adults may benefit the prevention and treatment of AD. We summarize in Figure 1 the potential mechanisms of interactions between the heart and the brain in AD and the associated intervention strategies.

Heart-brain interactions in AD are important for its prevention and treatment, and therefore we propose possible future research directions in this field. Further animal and clinical studies are needed to investigate the role of cardiac homeostasis and cardiovascular disease in the pathogenesis of AD. First, as most of the current studies on AD and cardiovascular health have been conducted in humans and fewer in animals, more work on animal models is also needed in the future, and given the significant differences between animals and humans, rhesus macaque and organoid models may be able to better explain the role of cardiac-derived substances in AD. Additionally, multiple animal models can be employed in research to gain a more comprehensive understanding, and comparisons should be made between animal experiments and clinical data to ensure the validity of the animal models to human diseases. Future research should focus on developing standardized models and combine these findings with clinical studies to ensure the translatability of experimental results. Second, this may lead to the generality of findings due to the heterogeneity of the study population, including different ethnicities between studies and some studies based on clinical populations. The methods of key studies may also have several limitations that affect the interpretation of results. Sample selection may introduce bias, potentially reducing the applicability of the results to a broader population. The design of the control group may not adequately reflect the conditions of the experimental group, which could impact the validity of the comparisons. Additionally, experimental conditions may not have strictly controlled potential confounding variables, raising questions about the reliability of the results. Caution is also needed when inferring causality in experimental studies, as correlation does not imply causation. Larger cohort studies are needed in the future to elucidate the causal relationship between cardiovascular disease and AD pathology as well as AD progression. Future research should also place greater emphasis on methodological rigor to ensure the validity and reliability of results. Third, current research primarily faces issues such as the lack of biological markers and limitations in treatment targets. Future studies can conduct large-scale prospective research utilizing genomics and proteomics to screen and validate new candidate biomarkers. Additionally, the exploration of new treatment targets should focus on mechanisms that have not yet been clearly defined. Fourth, the potential exploitation of heart-derived proteins as therapeutic targets for AD also needs to be validated in AD patients to translate findings from animal experiments into clinical studies. In addition, considering the complex crosstalk between the heart and brain, the identification of key molecules promoting AD in different cardiovascular diseases will be important for future therapies. Fifth, with advances in computer science, emerging technologies such as artificial intelligence and digital therapies are also highly likely to become new tools for combating AD due to heart-brain crosstalk. Furthermore, it advocates for an interdisciplinary research approach that combines cardiology, neurology, and geriatrics to address the complex interactions between cardiovascular health and AD. We hope that the present review will provide a springboard for further research on heart-brain crosstalk in AD. Thorough research in this area may help to improve patient's quality of life, reduce healthcare costs, and provide appropriate treatments for AD patients with comorbid cardiovascular disease.
